# ID 110 - Uso de TSH Recombinante em Adultos com Carcinoma Diferenciado de Tireoide e Contraindicação à Indução de Hipotireoidismo para Ablação com Radioiodo: revisão sistemática e metaanálise

**DOI:** 10.5327/2237-9622.2025.v34s1.110

**Published:** 2025-11-25

**Authors:** Isabela Pina Meza, Aline de Fátima Bonetti, Mariana Millan Fachi, Layssa Andrade Oliveira, Haliton Alves Oliveira, Rosa Camila Lucchetta

**Keywords:** neoplasias da glândula tireoide, tireotropina, TSH recombinante, qualidade de vida, revisão sistemática

## Abstract

**Introdução:**

O tratamento padrão para carcinoma diferenciado de tireoide (CDT) consiste na tireoidectomia total ou quase total, seguida de ablação com radioiodo no tecido tireoidiano remanescente. Para otimizar a eficácia da ablação, é necessário elevar os níveis de TSH, o que geralmente é obtido pela suspensão do hormônio tireoidiano por quatro a seis semanas após a tireoidectomia. No entanto, o hipotireoidismo induzido pela retirada da levotiroxina pode causar efeitos adversos significativos, levando à interrupção de algumas atividades de vida diária durante as semanas de hipotireoidismo, e consequente comprometimento da qualidade de vida (QV). Como alternativa, pode-se utilizar o TSH humano recombinante (rhTSH) antes da ablação. O rhTSH é uma fonte exógena de TSH que ativa o receptor de TSH, estimulando a captação de radioiodo no tecido tireoidiano remanescente, permitindo que o paciente continue o uso de levotiroxina no período pré-ablação. Assim, o objetivo deste estudo foi avaliar a eficácia e a segurança do uso do rhTSH em pacientes com CDT e contraindicação à indução de hipotireoidismo para ablação com radioiodo.

**Método:**

Foi realizada uma revisão sistemática de ensaios clínicos randomizados (ECR) identificados no PubMed, Embase e Cochrane Library, comparando o uso de rhTSH com a indução de hipotireoidismo em pacientes com CDT. Os desfechos avaliados incluíram taxa de ablação bem-sucedida, QV e segurança (eventos adversos gerais e graves). O risco de viés foi avaliado utilizando a ferramenta ROB 2.0, e a qualidade das evidências foi graduada conforme o sistema GRADE. Metanálise direta foi conduzida para taxa de ablação bem-sucedida.

**Resultados:**

Foram incluídos nove ECR que compararam o uso do rhTSH com a suspensão da levotiroxina. Não houve prejuízo no sucesso da taxa de ablação devido ao uso de rhTSH (RR 0,99 [IC 95% 0,96-1,02]; GRADE: moderada). Embora os resultados relacionados à QV fossem limitados, observou-se melhora precoce (até um mês após a ablação) no grupo tratado com rhTSH. A longo prazo, contudo, não foram identificadas diferenças significativas entre as intervenções (SF-36: componente físico variando de 38,5 a 73,4 vs 41 a 69,7, e componente mental de 40 a 52 vs 41 a 52, para intervenção e controle, respectivamente, com seguimento de quatro a seis meses; GRADE: muito baixa). Os relatos de eventos adversos (EAs) foram limitados, sem EAs graves relacionados aos tratamentos (GRADE: muito baixa), e o grupo rhTSH apresentou menor incidência de EAs gerais, como ganho de peso (RR: 0,57 [IC 95% 0,46-0,71]), movimentação lenta (RR: 0,24 [IC95%: 0,16-0,37]) e constipação (RR: 0,52 IC 95 [0,37-0,74]).

**Conclusão:**

A revisão não mostrou diferença significativa na taxa de ablação bem-sucedida entre a suspensão de levotiroxina e o uso de rhTSH em pacientes com CDT, com uma tendência de melhora precoce na QV no grupo tratado com rhTSH, não mantida a longo prazo. Em termos de segurança, o grupo rhTSH apresentou menor incidência de EAs, sem relatos de eventos graves. Esses resultados sugerem que o rhTSH é uma alternativa viável para pessoas com CDT, com potenciais benefícios a curto prazo em QV e menor ocorrência de EAs, sem comprometer a eficácia da ablação. No entanto, dado que a qualidade das evidências variou de baixa a moderada, há incerteza sobre a plausibilidade da substituição, sendo necessários novos estudos primários de alta qualidade para confirmar esses achados.

